# Osteoarthritic cartilage explants affect extracellular matrix production and composition in cocultured bone marrow-derived mesenchymal stem cells and articular chondrocytes

**DOI:** 10.1186/scrt466

**Published:** 2014-06-10

**Authors:** Michaela Leyh, Andreas Seitz, Lutz Dürselen, Hans-Robert Springorum, Peter Angele, Anita Ignatius, Joachim Grifka, Susanne Grässel

**Affiliations:** 1 Department of Orthopaedic Surgery, University of Regensburg, Asklepiosklinikum, Kaiser-Karl V.-Allee 3, 93077 Bad Abbach, Germany; 2 Centre for Medical Biotechnology, ZMB/BioPark I, Josef-Engert-Str. 9, 93053 Regensburg, Germany; 3 Institute of Orthopaedic Research and Biomechanics, Centre of Musculoskeletal Research, University of Ulm, Helmholtzstr 14, 89081 Ulm, Germany; 4 Department of Trauma Surgery, University of Regensburg, Franz-Josef-Strauss Allee 11, 93053 Regensburg, Germany

## Abstract

**Introduction:**

In the present study, we established a novel *in vitro* coculture model to evaluate the influence of osteoarthritis (OA) cartilage explants on the composition of newly produced matrix and chondrogenic differentiation of human bone marrow-derived mesenchymal stem cells (BMSCs) and the phenotype of OA chondrocytes. In addition, we included a “tri-culture” model, whereby a mixture of BMSCs and chondrocytes was cultured on the surface of OA cartilage explants.

**Methods:**

Gene expression analysis, protein and glycosaminoglycan (GAG) assays, dot-blot, immunofluorescence, and biomechanical tests were used to characterize the properties of newly generated extracellular matrix (ECM) from chondrocytes and chondrogenically differentiated BMSCs and a mix thereof. We compared articular cartilage explant cocultures with BMSCs, chondrocytes, and mixed cultures (chondrocytes and BMSCs 1:1) embedded in fibrin gels with fibrin gel-embedded cells cultured without cartilage explants (monocultures).

**Results:**

In general, co- and tri-cultured cell regimens exhibited reduced mRNA and protein levels of collagens I, II, III, and X in comparison with monocultures, whereas no changes in GAG synthesis were observed. All co- and tri-culture regimens tended to exhibit lower Young’s and equilibrium modulus compared with monocultures. In contrast, aggregate modulus and hydraulic permeability seemed to be higher in co- and tri-cultures. Supernatants of cocultures contained significant higher levels of interleukin-1 beta (IL-1β), IL-6, and IL-8. Stimulation of monocultures with IL-1β and IL-6 reduced collagen gene expression in BMSCs and mixed cultures in general but was often upregulated in chondrocytes at late culture time points. IL-8 stimulation affected BMSCs only.

**Conclusions:**

Our results suggest an inhibitory effect of OA cartilage on the production of collagens. This indicates a distinct modulatory influence that affects the collagen composition of the *de novo*-produced ECM from co- and tri-cultured cells and leads to impaired mechanical and biochemical properties of the matrix because of an altered fibrillar network. We suggest that soluble factors, including IL-1β and IL-6, released from OA cartilage partly mediate these effects. Thus, neighbored OA cartilage provides inhibitory signals with respect to BMSCs’ chondrogenic differentiation and matrix composition, which need to be accounted for in future cell-based OA treatment strategies.

## Introduction

Therapeutic approaches in musculoskeletal regenerative medicine which use adult bone marrow-derived mesenchymal stem cells (BMSCs) are promising as they facilitate tissue repair and regeneration. BMSCs are superior to differentiated cells (that is, chondrocytes) as they are well expandable *in vitro* and retain their pluripotency over several passages. BMSCs are known to easily differentiate into mesenchymal lineages *in vitro*, including cartilage, bone, and adipose tissue, after appropriate induction [1,2]. The regulation of chondrogenic differentiation of BMSCs and the production of a typical hyaline articular cartilage extracellular matrix (ECM) involve a variety of factors: stem cell intrinsic factors, paracrine factors from neighboring cells like osteoblasts or chondrocytes, and yet poorly defined additional microenvironmental components. Independent from cells, chondrogenic differentiation of BMSCs and deposition of new ECM can be induced by stimulation with growth factors like bone morphogenic proteins (BMPs) or other transforming growth factor-beta (TGF-β) superfamily members [2-4]. Interaction between BMSCs and ECM components provides an instructive microenvironment and contributes to differentiation of BMSCs into chondrocytes, suggesting a beneficial effect in using BMSCs in conjunction with synthetic or natural scaffolds which provide a three-dimensional (3D) environment compared with cells alone [5,6]. A commonly used hydrogel scaffold is based on fibrin (fibrin gel or fibrin glue) that encapsulates BMSCs or chondrocytes and mimics the structure and function of a natural ECM by maintaining a round morphology of cells and thus facilitating deposition of new ECM components [7,8]. ECM components are strongly correlated to mechanical strength of engineered cartilage-like tissue. Key roles in biomechanical properties have—among other roles— correctly deposited and interconnected proteoglycans and collagens [9,10]. Proteoglycans which form the extrafibrillar matrix and collagen fibrils which form the fibrillar matrix determine the level of hydration and stability of the ECM and therefore the mechanical properties of articular cartilage. Therefore, the glycosaminoglycan (GAG) and collagen contents are useful for prediction of mechanical tissue properties. Thus, efficacy and the ability of proper differentiated BMSCs to produce a highly organized functional ECM are important for the quality and integrity of regenerated tissue [11]. The differentiation potential of BMSCs implanted into trauma-induced focal articular cartilage defects and their ability to produce a proper and stable ECM which integrates closely with the adjacent healthy tissue are highly dependent on the response of the BMSCs to the tissue microenvironment provided by cell-cell and cell-matrix interaction and factors released from the neighboring tissue [5,9].

The ability of BMSCs to differentiate into a specific cell phenotype therefore is critically dependent on the surrounding environment. Recent studies have demonstrated the ability of paracrine factors released by healthy cartilage tissue or articular chondrocytes to induce chondrogenesis of progenitor cells. In addition, a healthy articular cartilage microenvironment enhances the chondrogenic differentiation capacity of BMSCs and leads to a higher collagen and GAG content in the ECM while preventing hypertrophic differentiation [12,13]. However, the effect of diseased cartilage and osteoarthritis (OA) chondrocytes on chondrogenic differentiation of BMSCs is poorly understood. OA chondrocytes secrete factors, such as pro-inflammatory cytokines and chemokines, that are believed to have a negative effect on locally residing progenitor cells and inhibit the cartilage repair *in vivo*[14].

To study the effects of direct cell-cell contact, many coculture studies investigated cellular interactions between different cell types—that is, mesenchymal stem cells (MSCs) and chondrocytes—by culturing the cells together in mixed 3D micromass pellets in different ratios [13,15-18]. To analyze the effects of paracrine soluble factors derived from cocultured cells, other coculture studies investigated the effect of two types of cells, which were separated (that is, through porous membranes) [12,19-22]. However, none of these studies used a coculture system, where at least one cell type was kept in its original 3D matrix environment. Also, none of the studies investigated the effects of direct cellular interaction between two cell types and response to soluble factors from intact cartilage tissue explants in parallel. We designed a novel coculture model which includes explants from articular cartilage derived from OA patients cocultured with cells embedded in fibrin gel. With that model, we examined the effects of paracrine factors from articular cartilage explants on BMSCs differentiation and phenotype of differentiated chondrocytes and the properties of their newly formed ECM. The advantage of using explants instead of isolated chondrocytes is that effects of the original cartilage ECM are still present and this is crucial for future cell-based regenerative approaches of cartilage defects. In addition, we introduced a third culture condition in which we cocultured a mixed population of BMSCs and chondrocytes (1:1) embedded in fibrin gel with cartilage explants. With that “tri-culture” model, we aimed to study combined paracrine and cell-cell effects.

## Materials and methods

### Culture and isolation of human articular cartilage explants, bone marrow-derived stem cells, and chondrocytes

Human articular cartilage pieces were collected from surgically removed joints of patients undergoing total knee replacements due to OA. Rare healthy cartilage was obtained from knee surgeries due to traumatic accidents. This had been approved by the local ethics committee (Az: 08/065; Ethikkommission an der Universität Regensburg), and specimens were taken with patients’ written consent. For this study, knee joints were obtained from different donors, and about 30 different sets of cartilage explants of similar OA grade were prepared. Prior to culture, cartilage tissue was first classified macroscopically as either damaged or intact according to a predefined procedure comprising color, surface integrity, and tactile impression tested with a standard scalpel [23]. For the coculture setup, cartilage chips were punched out (8 × 2 mm) from intact cartilage slices including the superficial zone. Chondrocytes were isolated from cartilage slices as described elsewhere [21], cultured for 7 to 14 days, and used when confluent (passage 1).

**Figure 1:**
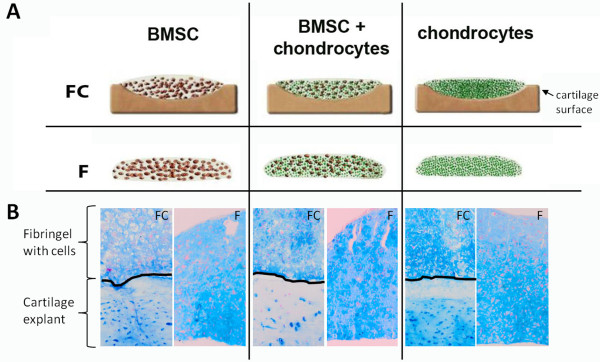
**Cell culture models. (A)** Bone marrow-derived stem cells (BMSCs), mixed cultures (BMSCs and chondrocytes in a ratio of 1:1), and chondrocytes were embedded in fibrin gels and applied onto the surface of osteoarthritis articular cartilage explants (co- and tri-cultures). As controls, cells were embedded in fibrin gels and cultured without cartilage (monocultures). All experimental setups were kept for up to 28 days in chondrogenic medium. Samples were harvested at days 7 and 28. Chondrocytes, BMSCs, and cartilage explants were not used autologous, and each “N” represents different donors. **(B)** Representative overview of alcian blue stained fibrin gels of BMSCs, mixed cultures (BMSCs + chondrocytes 1:1), and chondrocytes after 28 days of mono-, co-, or tri-culture with osteoarthritis cartilage. F, monocultures (without cartilage explants); FC, co- and tri-cultures with cartilage explants.

Human BMSCs have been isolated from bone marrow aspirates obtained from patients undergoing hip replacement surgery. This had been approved by the local ethics committee (Az: 08/065; Ethikkommission an der Universität Regensburg), and specimens were taken with patients’ written consent. BMSCs from a total of 30 different bone marrow donors were included in this study. The bone marrow was centrifuged, and cells were fractionated on a density gradient (Biocoll Separating Solution; Biochrom, Berlin, Germany). The low-density cell fraction concentrated in the interphase (“buffy coat”) was washed and seeded in cell culture flasks supplied with MesenchymStem Medium (PAA, Piscataway, NJ, USA), and non-adherent cells were removed after 5 to 7 days. Adherent cells were cultured until they reached approximately 80% confluence. After splitting, BMSCs were seeded at a density of 4 × 10^4^ cells per cm^2^ and kept in culture for up to three passages before they were used for experiments. BMSCs were shown to be positive for CD44 and CD105 and negative for CD19 and CD34 and were capable of differentiating into osteogenic, adipogenic, and chondrogenic lineages (data not shown). A suspension of fibrinogen (10 μL, 100 mg/mL; Sigma-Aldrich, St. Louis, MO, USA) and 1 × 10^6^ BMSCs, 2 × 10^6^ chondrocytes, or a mixture of 5 × 10^5^ BMSCs and 5 × 10^5^ chondrocytes (1:1) was homogenously mixed with thrombin (18 μL, 5 U/mL; Baxter, Munich, Germany). The cell-fibrinogen suspension was applied on the surface of the superficial zone of articular cartilage explants or as a droplet on the bottom of a 24-well plate (Figure 1). Notably, cartilage explants, chondrocytes, and BMSCs were not used autologous. Therefore, in the mixed cultures, up to three different patients were merged. Full polymerization of the suspension was reached after 45 minutes at 37°C and resulted in a stable and clear hydrogel with a pore size of approximately 50 μm. Cell-free cartilage explants were used as controls. Co-, tri-, and monocultures as well as cell-free cartilage explants were kept in chondrogenic medium in the presence of TGFβ-3 (10 ng/mL; R&D Systems, Minneapolis, MN, USA) [12] and cultured for up to 4 weeks. After 7 and 28 days, fibrin gels were processed for histology, immunofluorescence, protein biochemistry, biomechanics, or gene expression analysis as described below. Supernatants of days 7 and 28 were collected and frozen at -80°C until they were used for enzyme-linked immunosorbent assay (ELISA), hydroxyproline assay, and dimethylmethylene blue (DMMB) assay or collagen preparation.

### Stimulation of bone marrow-derived stem cells or chondrocytes (or both) embedded in fibrin gels

Stimulation of monocultured BMSCs, mixed cell cultures, and chondrocytes embedded in fibrin gel with interleukin-1 beta (IL-1β) (5 ng/mL; Biomol, Hamburg, Germany), IL-6 (5 ng/mL; RayBiotech, Norcross, GA, USA), or IL-8 (10 ng/mL; RayBiotech) was performed in chondrogenic medium for the first 7 days. After 7 and 28 days, fibrin gels were processed for gene expression analysis as described below. In total, seven independent stimulation setups for each interleukin were analyzed in triplicate (n = 7).

### Cell vitality and proliferation in fibrin gel cultures

To evaluate vitality of cells in our different culture setups, lactate dehydrogenase (LDH) concentration in supernatants was analyzed at days 7, 14, 21, and 28 with an LDH-based cytotoxicity detection Kit (Roche, Penzberg, Germany) in accordance with the instructions of the manufacturer. LDH concentration released from dead cells into supernatant was determined on a photometrical basis at an absorption of 490 nm (Tecan GENios with Magellan 6.5; Tecan, Crailsheim, Germany). In total, four independent coculture setups were analyzed in triplicate (n = 4).

For proliferation tests, fibrin gel-embedded cultures were processed as described under the section "Histology and immunofluorescence of fibrin gel-embedded cultures". Briefly, sections were fixed in 4% paraformaldehyde (PFA) (Sigma-Aldrich), and endogenous peroxidase was blocked by treatment with 3% H_2_O_2_ containing 10% EtOH followed by epitope demasking (boiling with 10 mM sodium citrate containing 0.05% Tween). After blocking, slides were subsequently incubated with an antibody against proliferating cell nuclear antigen (PCNA) (Dako, Glostrup, Denmark; mouse anti-PCNA clone PC10-M0879), a biotinylated secondary antibody (Dako), and streptavidin peroxidase. Staining was performed with the liquid substrate system (Dako) and counterstained with Mayer’s hemalaun solution (Roth, Arlesheim, Germany). Stained sections were photographed with an Olympus BX 61 imaging system (Olympus, Hamburg, Germany) and cell^P^ software (Olympus), the number of total nuclei and number of PCNA-positive stained nuclei were counted, and the proliferation ratio was calculated. In total, three independent stimulation setups were analyzed by counting at least three different sections (n = 3).

### Histology and immunofluorescence of fibrin gel-embedded cultures

Fibrin gels were rinsed with phosphate-buffered saline (PBS) (PAA), embedded in TissueTec (Sakura Finetek, Alphen aan den Rijn, The Netherlands), and frozen in liquid nitrogen. For histology, sections (10 μm thick) were fixed in 4% PFA (Sigma-Aldrich) for 10 minutes at room temperature (RT) and stained with 1% Alcian blue 8GX (Sigma-Aldrich) for GAGs and counterstained with nuclear fast red aluminum sulfate solution (Roth). For immunofluorescence analysis, sections were fixed in 4% PFA for 10 minutes at RT, treated for 30 minutes at 37°C with pepsin (3 mg/mL in 0.01 M HCl; Sigma-Aldrich), and blocked with 3% bovine serum albumin (Sigma-Aldrich) diluted in PBS for 1 hour. Sections were labeled overnight at 4°C with an antibody against collagen I (C-2456; Sigma-Aldrich), collagen II (CIIC1; Developmental Studies Hybridoma Bank, Iowa City, IA, USA), or collagen III (MAB3392; Merck Millipore, Billerica, MA, USA). In the case of collagen X staining (1-CO100-05; Quartett, Berlin, Germany), a hyaluronidase (Sigma-Aldrich) pretreatment instead of pepsin was used. For fluorescence detection, all sections were incubated with an Alexa 488 secondary antibody (Invitrogen, Paisley, UK) for 1 hour at 37°C. Finally, samples were mounted in Vectashield mounting medium with 4′,6-diamidino-2-phenylindole (DAPI) (1 μg/mL; Vector Laboratories, Burlington, ON, Canada) and were analyzed with an Olympus BX 61 imaging system and cell^P^ software. At least five culture setups (different donors) were analyzed in triplicate, and representative slides were chosen by two observers (n = 5).

### Biochemical analysis of fibrin gel cell lysates and culture supernatants

Fibrin gels were removed from cartilage explants, homogenized, and digested with pepsin (1 mg/mL in 0.5 M acetic acid containing 0.4 M NaCl; Sigma-Aldrich) for 48 hours at 4°C and further digested with elastase (1 mg/mL in Tris-buffered saline (TBS) pH 8; Serva Electrophoresis, Heidelberg, Germany) for 24 hours at 4°C. Samples were stored at -20°C until they were analyzed for GAGs and collagens. At least seven culture setups (different donors) were analyzed in triplicate (n = 7).

GAG concentration was measured spectrophotometrically by using 25 μL of the digested cell lysates or 25 μL of the undiluted cell culture supernatant supplemented with DMMB (AppliChem, Darmstadt, Germany) which forms a complex with GAGs. Quantification was performed in micrograms per 1 × 10^6^ cells with a chondroitin sulfate standard at 525 nm (Tecan GENios with Magellan 6.5; Tecan).

Collagen I and II contents of day 28 cell lysates digested as described above were measured with specific sandwich ELISAs which recognize the native conformation of collagen I and II chains (Chondrex, Inc., Redmond, WA, USA) in accordance with the instructions of the manufacturer. Collagen III in digested cell lysates was quantified with a dot-blot assay using an aliquot of 1 μL from 500 μL of total lysates using a standard curve (Abcam, Cambridge, UK). Quantification was performed densitometrically in micrograms per 1 × 10^6^ cells using a Chemi-Smart 500 (PeqLab, Erlangen, Germany) for fluorescence detection and the CS4 Windows software for calculation of luminescence intensities.

### RNA isolation and real-time polymerase chain reaction amplification

After suspension of cell-fibrin gels in peqGOLD TriFast (PeqLab), they were minced and RNA was isolated according to a Trizol protocol, followed by column purification with the absolutely RNA Microprep Kit (Agilent Technologies Stratagene, Santa Clara, CA, USA) in accordance with the instructions of the manufacturer. cDNA was generated from 500 ng of RNA by using an Affinity Script qPCR cDNA synthesis Kit and oligo(dT) primers (Agilent Technologies Stratagene) in accordance with the instructions of the manufacturer. At least six culture setups (different donors) were analyzed in triplicate (n = 6).

Quantitative real-time polymerase chain reaction (PCR) was performed in triplicate by using 30 ng cDNA and qPCR master mix SYBR Green Dye I on MxPro-Mx305P (Agilent Technologies Stratagene). For quantification, a plasmid standard curve was included on each PCR plate (plasmid copy ranges for *COL1A1*: 1 × 10^6^ to 1 × 10^2^; *COL2A1*: 3.4 × 10^7^ to 3.4 × 10^3^; *COL3A*1: 1 × 10^6^ to 1 × 10^2^; and *COL10A1*: 5 × 10^4^ to 5 × 10°). Data analysis was carried out by using MxPro-Mx305P QPCR 4.0 software (Agilent Technologies Stratagene). The following forward and reverse primer pairs were used for gene expression analysis: for *COL1A1* 5′-AGC TCC TGG TGA AGT TGG TC-3′ and 5′-ACC AGG GAA GCC TCT CTC TC-3′, for *COL2A1* 5′-TGC TGC CCA GAT GGC TGG AAG A-3′ and 5′-TGC CTT GAA ATC CTT GAG GCC C-3′, for *COL3A1* 5′-GTC CAT GGA TGG TGG TTT TC-3′ and 5′-GTG TGT TTC GTG CAA CCA TC-3′, and for *COL10A1* 5′-CCC TCT TGT TAG TGC CAA CC-3′ and 5′-AGA TTC CAG TCC TTG GGT CA-3′.

### Analysis of soluble collagens in culture supernatants

The amount of total soluble collagen in culture supernatants was determined by the Total Collagen Hydroxyproline Assay in accordance with the protocol of the manufacturer (QuickZyme Biosciences, Leiden, The Netherlands). Briefly, 1 mL of culture supernatant was removed after 3 days of culture, and soluble collagens in the supernatant were hydrolyzed into amino acids (12 M HCl for 20 hours at 95°C). Hydroxyproline was stained, and color formation was quantified at 570 nm (Tecan GENios with Magellan 6.5; Tecan). At least seven culture setups (different donors) were analyzed in triplicate (n = 7).

### Analysis of culture supernatants for interleukin (IL)-1β, IL-6, and IL-8

To determine the concentration of specific proteins in the supernatant, the human IL-1β sandwich ELISA kit (RayBiotech), IL-6 sandwich ELISA kit (R&D Systems), and IL-8 ELISA Kit (Gen-Probe, now part of Hologic, Bedford, MA, USA) were used in accordance with the instructions of the manufacturers. At least seven culture setups (different donors) were analyzed in triplicate (n = 7).

### Biomechanical testing

After removal from cartilage explants, all fibrin gel cell constructs were cut to pieces of the same size by punching out constructs with an outer diameter of 2.6 mm (biopsy punch; Stiefel GmbH, München, Germany). All biomechanical tests were carried out in a standard material testing machine (Z010; Zwick GmbH, Ulm, Germany) using a 40 N load cell. The initial height (h_0_) was measured under a preload of 0.1 N using a laser displacement transducer (optoNCDT 2200-20; Micro-Epsilon GmbH & Co. KG, Ortenburg, Germany; 0.3 μm resolution, ±0.03% accuracy). An unconfined compression test was performed by placing the samples in a cell culture dish filled with 0.9% NaCl and loading it by a flat-ended cylinder at a strain rate of 100% h_0_/minute until 50% strain was reached. The Young’s modulus was determined from the related stress-strain diagrams. Two typical regions were evaluated: the progressive region at 0% to 10% strain and the linear region at 40% to 50% strain. After adequate relaxation time of 24 hours, an additional relaxation test was performed under confined compression conditions. The samples were placed in a confining chamber (2.6 mm in diameter) filled with 0.9% NaCl and loaded by a flat-ended porous ceramic cylinder (Al_2_O_3_) allowing fluid flow. After application of 50% strain at a strain rate of 100% h_0_/minute, the strain was constantly held over a time of 10 minutes until the equilibrium state was reached. On the basis of these data, hydraulic permeability (k) was calculated referred to a given diffusion equation [24,25] using Formula 1. The aggregate modulus (H_A_) at equilibrium state (50% strain) was assessed using Formula 2 considered that Δl/h_0_ is the applied strain, H the modulus and σ_∞_ the stress at equilibrium state.

(1) $$\sigma_{t}=\sigma_{\infty }+2H*\frac{\mathrm{\Delta l}}{h_{o}}*e^{\left({\left(\frac{\pi }{h_{o}}\right)}^{2}*H*K*t\right)}$$

(2) $$H_{A}=\frac{\sigma_{\infty }}{\varepsilon_{50\%}}$$

### Statistical analysis

The mean standard deviation values were calculated for all variants. The non-parametric Wilcoxon test (for paired analyses) or the Mann-Whitney test (unpaired analyses) was applied to analyze differences between time points and between culture conditions. All experiments were repeated at least five times with cells from different donors. *P* values of less than 0.05 were considered to indicate statistically significant differences. Owing to the limited sample number provided for the biomechanical tests, these data were analyzed descriptively. Data analysis and graphing were performed with GraphPad for Windows version 5 (GraphPad Software Inc., San Diego, CA, USA).

## Results

### Proliferation and viability

To evaluate whether the fibrin gel system affects vitality/viability of cells, LDH concentration in culture supernatants was analyzed and compared with respective monolayer controls. Chondrocytes, BMSCs, and mixed cultures embedded in fibrin gel cocultured or kept as monocultures showed no enhanced LDH release compared with assay controls. We therefore assume that cells are not influenced in their vitality by fibrin gel components or cartilage explants during the culture time period (Additional file 1: Figure S1A-C).

Fibrin gel-embedded cultures were stained with PCNA in order to determine whether cells proliferate. In all culture conditions, positive PCNA staining was detected, indicating mitotic activity. Counting of PCNA stained cell nuclei revealed no significant differences in mitotic activity of tri- and cocultured cells compared with monocultured cells during the entire culture period (Additional file 1: Figure S1D).

Coculture conditions with cartilage explants and fibrin gel components did not affect mitotic activity or vitality of BMSCs, mixed cultures, or chondrocytes.

### Culture models

We have set up different culture conditions to analyze the influence of factors from cartilage tissue on multipotent BMSCs with respect to ECM formation and chondrogenic differentiation. Therefore, we have cocultured BMSCs embedded in fibrin gel with cartilage explants from patients with OA. As a control for chondrogenic properties of BMSCs, we have used cocultures of differentiated articular chondrocytes as well as mixed cultures (tri-cultures) of chondroctyes and BMSCs (in a ratio of 1:1) to include putative cell-to-cell effects of differentiated cells on undifferentiated cells. As a control for cocultures, we have set up the respective monocultures (cultured without cartilage explants) of cells embedded in fibrin gels and cultured in chondrogenic medium (Figure 1A). Alcian blue staining was used to detect proteoglycan/aggrecan deposition in the fibrin gel constructs. All culture conditions were nicely stained blue and showed no differences between monocultures and cartilage co- and tri-cultures (Figure 1B).

### Collagen gene expression of co- and tri-cultures versus monocultures

**Figure 2:**
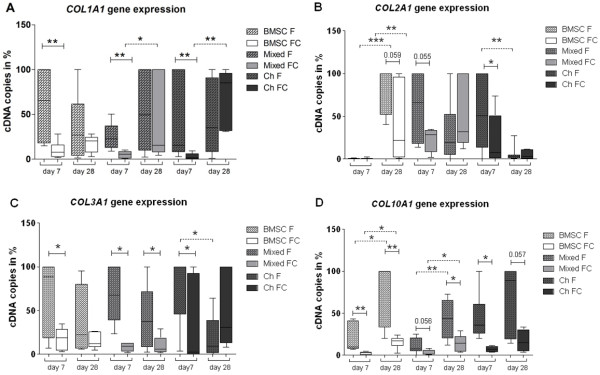
**Quantification of gene expression with quantitative polymerase chain reaction.** Gene expression levels of **(A)***COL1A1*, **(B)***COL2A1*, **(C)***COL3A1*, and **(D)***COL10A1* were determined in mono-, co-, and tri-cultures by using standard curves. Bone marrow-derived stem cells (BMSCs) (white bars), mixed cultures (BMSCs and chondrocytes in a ratio of 1:1, light grey bars), or chondrocytes (dark grey bars) were kept as monocultures (F, bars with pattern) or as co- and tri-cultures with osteoarthritis cartilage explants (FC, solid bars) in chondrogenic medium. Owing to high inter-experimental variability, we have calculated the raw data as percentage of highest cDNA copy number per individual experiment. Results are presented as mean with standard deviation. *P <0.05; **P <0.01; ***P <0.001. N = 6. Ch, chondrocytes.

To determine whether OA cartilage explants affect gene expression of ECM macromolecules in BMSCs and chondrocytes, mRNA expressions of *COL1A1* (de-differentiation marker), *COL2A1* (chondrogenic differentiation marker), *COL3A1* (mesenchymal cell marker), and *COL10A1* (hypertrophic chondrocyte marker) were analyzed at days 7 and 28 by using qPCR. At day 7, we observed a significant inhibition of *COL1A1* gene expression in all cocultured regimens in comparison with monocultures. An increase of *COL1A1* gene expression was detected in mixed and chondrocyte cocultures from day 7 to 28 (Figure 2A). *COL2A1* gene expression on day 7 was significantly downregulated in mixed tri- and chondrocyte cocultures and on day 28 was significantly decreased in BMSC cocultures compared with monocultures. In BMSC mono- and cocultures, we detected a highly significant upregulation of *COL2A1* gene expression from day 7 to 28, suggesting induction of chondrogenic differentiation. *COL2A1* gene expression in chondrocyte cultures was significantly downregulated from day 7 to 28 of culture (Figure 2B). *COL3A1* gene expression was significantly reduced in mixed tri-cultures over the entire culture period and in BMSC and chondrocyte cocultures at day 7 compared with monocultures. A significant downregulation of *COL3A1* gene expression from day 7 to 28 was detected in chondrocyte monocultures (Figure 2C). *COL10A1* gene expression was inhibited in all coculture setups which was significant at days 7 and 28 in comparison with monocultures. In BMSCs and mixed mono- and cocultures, we observed significant upregulation of C*OL10A1* gene expression from day 7 to 28 (Figure 2D).

Overall, collagen gene expression in cocultured BMSCs and chondrocytes and mixed tri-cultures was significantly reduced compared with monocultures.

### Quantification of collagen and glycosaminoglycan production

**Figure 3:**
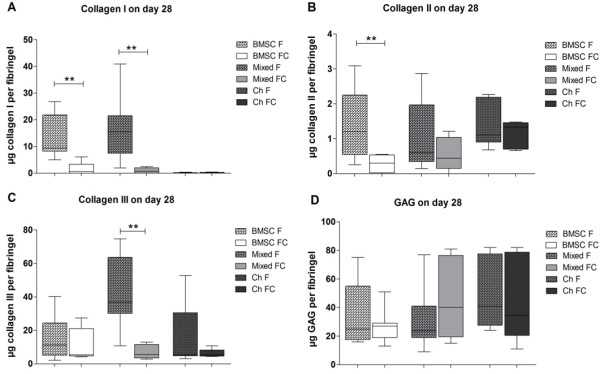
**Quantification of collagens I, II, and III and glycosaminoglycan (GAG) in fibrin gel lysates.** Protein synthesis of collagens **(A)** I, **(B)** II, **(C)** III, and **(D)** GAG in cell lysates after 28 days of culture. Bone marrow-derived stem cells (BMSCs) (white bars), mixed cultures (BMSCs and chondrocytes in a ratio of 1:1, light grey bars), or chondrocytes (dark grey bars) were kept as monocultures (F, bars with pattern) or as co- and tri-cultures with osteoarthritis cartilage explants (FC, blank bars) in chondrogenic medium. **(A, B)** Collagens I and II were quantified with enzyme-linked immunosorbent assay or **(C)** for collagen III by densitometrically evaluated dot-blot analysis including a recombinant collagen III standard curve. **(D)** GAG concentration in cell lysates was quantified by a dimethylmethylene blue assay including a chondroitin sulfate standard curve. Results are presented as mean with standard deviation. *P <0.05; **P <0.01. N = 7. Ch, chondrocytes.

Next, we studied the influence of cartilage explants on collagen and proteoglycan synthesis/degradation. Collagen I protein was found to be synthesized in BMSCs and mixed monocultures at the microgram level but not in chondrocyte mono- and cocultures. A collagen I-specific ELISA revealed a significantly reduced collagen protein content for BMSC co- and mixed tri-cultures in comparison with monocultures (Figure 3A). A collagen II-specific ELISA revealed that collagen II synthesis in BMSCs was clearly inhibited by cartilage explant coculture in comparison with monocultures. For mixed tri-cultures and cocultured chondrocytes, we did not detect significant differences between both culture conditions (Figure 3B). Dot-blot analysis of collagen III protein content demonstrated a significant reduction in mixed tri-cultures, whereas no significant differences were detectable in BMSC or chondrocyte cocultures versus monocultures (Figure 3C). In contrast to the collagenous ECM components, GAG content in BMSC, mixed culture, and chondrocyte cell lysates was not affected by coculture (Figure 3D).

Overall, we detected a decrease in collagen I, II, and III concentration in cocultured BMSCs and mixed tri-cultured lysates but not in chondrocyte cocultures. We did not observe an inhibitory effect on GAG synthesis in co-and tri-cultures.

### Immunofluorescence staining of collagens

**Figure 4:**
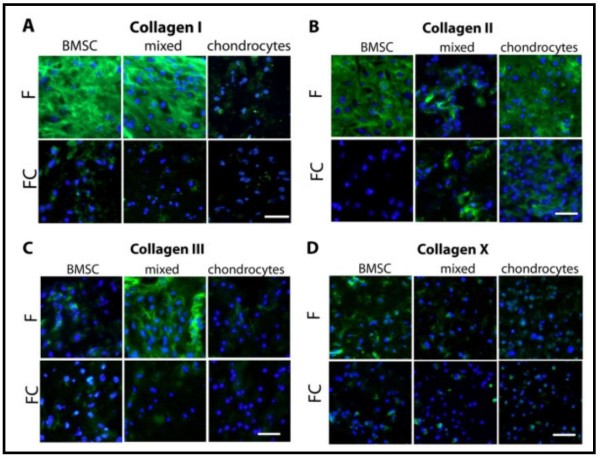
**Immunofluorescence staining of collagens.** Representative immunofluorescence staining images of collagens **(A)** I, **(B)** II, **(C)** III, and **(D)** X detected on cryosections after 28 days of culture. Bone marrow-derived stem cells (BMSCs), mixed cultures (BMSCs and chondrocytes in a ratio of 1:1), or chondrocytes were monocultured (F) or co-/tri-cultured with osteoarthritis cartilage (FC). Nuclei were stained with 4′,6-diamidino-2-phenylindole (DAPI) (blue color). Scale bar is 100 μm. N = 5.

During *de novo* production and deposition of ECM, the cell-fibrin gel constructs changed their color from translucent to milky opaque. However, they kept their lense shape during the entire culture period, and no shrinking of constructs was observed. ECM deposition of collagens from BMSCs, mixed cultures, and chondrocytes in mono-, co-, and tri-culture conditions was evaluated by immunofluorescence staining of cryosections at day 28. Monocultured BMSC and mixed cultures deposited a higher amount of collagen I into their ECM compared with co- and tri-cultures. Chondrocytes revealed only little collagen I staining in both culture conditions (Figure 4A). The presence of collagen II was demonstrated in BMSC monocultures, but no staining was detected in cocultures. Mixed cultures showed similar collagen II staining in both culture conditions. Chondrocyte monocultures exhibited a strong collagen II signal in their ECM comparable to BMSC monocultures with somewhat reduced staining in cocultures (Figure 4B). Collagen III deposition in chondrocytes and BMSCs remained unchanged by coculture with cartilage. Tri-cultures of mixed cells revealed a weaker collagen III signal compared with monocultures (Figure 4C). Deposition of collagen X was detected in BMSC monocultures. In all other culture regimens, there was only poor collagen X staining (Figure 4D).

Overall, immunofluorescence staining of ECM deposited collagens I, II, III, and X supported data of collagen quantification with ELISA and dot blot.

### Biomechanical properties

**Figure 5:**
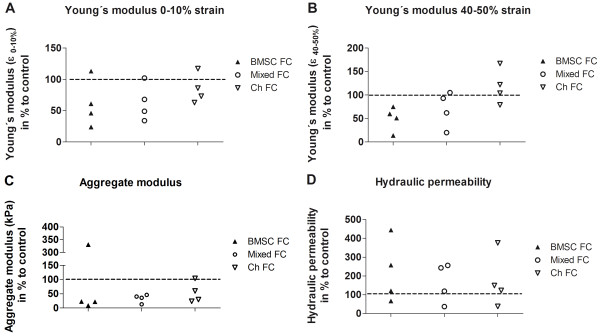
**Biomechanical properties.** Biomechanical properties of the newly formed extracellular matrix at day 28 of bone marrow-derived stem cells (BMSCs), mixed cultures, or chondrocytes co- and tri-cultured with osteoarthritis cartilage (FC) were analyzed and calculated in relation to monocultured controls (dotted lines at 100% represent monoculture values). Young’s modulus of **(A)** 0% to 10% and **(B)** 40% to 50% strain was determined by using unconfined compression. Aggregate modulus at equilibrium **(C)** and hydraulic permeability **(D)** were determined by using confined compression performed at 50% compressive strain. Ch, chondrocytes.

We determined mechanical properties and load capacity of newly generated ECM. Unconfined mechanical testing indicated that cocultured BMSCs, chondrocytes, or a mixture of both cell types exhibited a decrease in Young’s modulus (0% to 10% strain) in 3 out of 4 samples of each cell type (Figure 5A) and a decrease in 4 out of 4 samples (BMSCs) or 3 out of 4 samples (mixed cultures) at 40% to 50% strain (Figure 5B). In cocultured chondrocytes, Young’s modulus under 40% to 50% strain tended to be higher in 3 out of 4 samples (Figure 5B). Aggregate modulus at equilibrium was lowered in almost any cases for all co- and tri-cultured samples (Figure 5C). Hydraulic permeability of cartilage co- and tri-cultures was increased in comparison with monocultures in 3 out of 4 samples (Figure 5D).

Overall, biomechanical tests showed that ECM in monocultured BMSC and mixed fibrin gel constructs seemed to be stiffer and less porous and thus in general of higher integrity and stability—with respect to Young’s modulus, aggregate modulus, and hydraulic permeability—than newly formed matrix in co- and tri-cultures. Mechanical parameters in chondrocyte cocultures were partly superior to monocultures.

### Quantification of cytokines and glycosaminoglycan in culture supernatants

**Figure 6:**
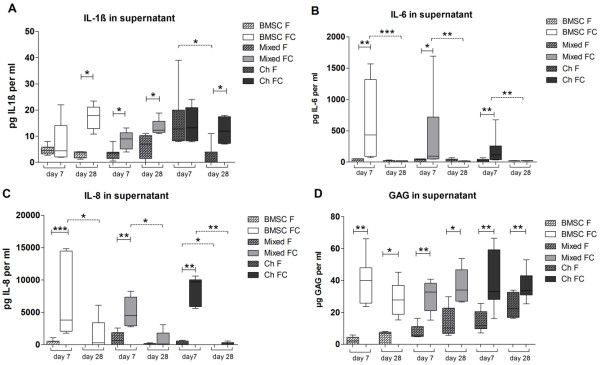
**Quantification of cytokines and glycosaminoglycan (GAG) in culture supernatants.** Analysis of supernatants at days 7 and 28 of bone marrow-derived stem cells (BMSCs) (white bars), mixed cultures (BMSCs and chondrocytes in a ratio of 1:1, light grey bars), or chondrocytes (dark grey bars) monocultured (F, bars with pattern) or co- tri-cultured with osteoarthritis cartilage (FC, blank bars). Total amount of cytokines **(A)** interleukin-1 beta (IL-1β), **(B)** IL-6, and **(C)** IL-8 was quantified by antigen-specific enzyme-linked immunosorbent assays. **(D)** GAG released into the supernatant was quantified by dimethylmethylene blue (DMMB) assay. Results are presented as mean with standard deviation. *P <0.05; **P <0.01; ***P <0.001. N = 6. Ch, chondrocytes.

**Figure 7:**
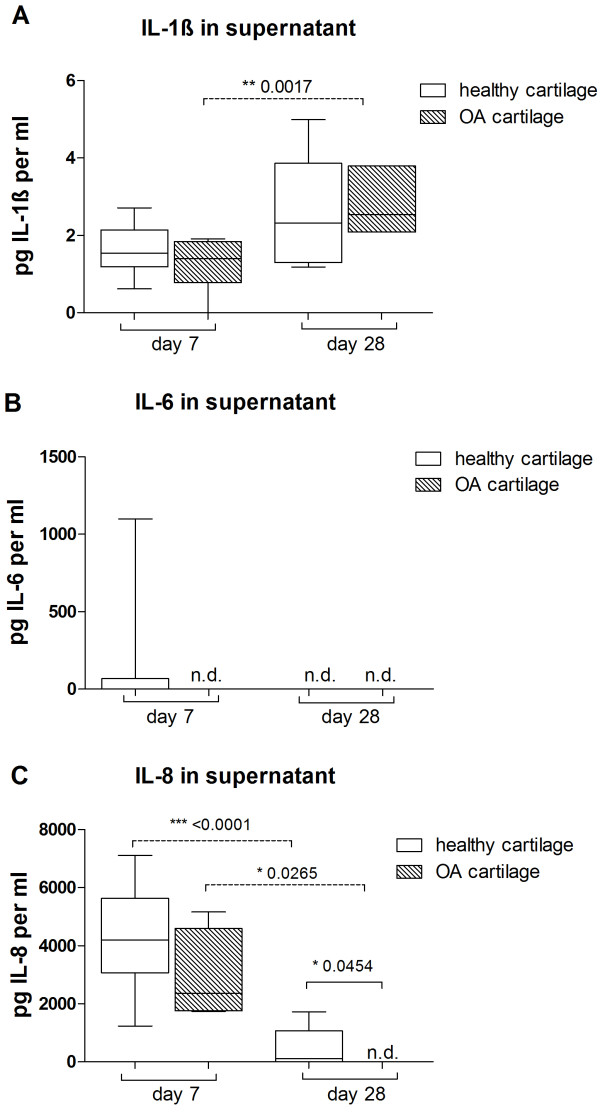
**Quantification of cytokines in cell-free cartilage explant supernatants.** Concentrations of **(A)** interleukin-1 beta (IL-1β), **(B)** IL-6, and **(C)** IL-8 were determined in cell-free healthy (white bars) and osteoarthritis (OA) cartilage (grey bars) explant cultures kept in chondrogenic medium for 7 or 28 days. Results are presented as mean with standard deviation. *P <0.05; **P <0.01; ***P <0.001; N = 5. n.d., not determined.

To quantify representative pro-inflammatory cytokines released into culture supernatants, we performed ELISAs for IL-1β, IL-6, and IL-8. Results revealed that BMSCs, mixed cultures, and chondrocytes cocultured with cartilage explants secrete significant higher IL-1β levels at day 28 than monocultures. At day 7, only mixed tri-cultures secreted significant higher IL-1β levels (Figure 6A). Furthermore, cocultured BMSCs, mixed cultures, and chondrocytes had significantly elevated IL-6 and IL-8 levels at day 7 compared with monocultures. Over the culture time, IL-6 and IL-8 levels decreased significantly from day 7 to 28 in supernatants of cocultures (Figure 6B and C). In addition, we tested supernatants of explants cultured without cells, where we detected low levels of IL-1β for OA cartilage (1.1 ± 0.7 pg/mL at day 7 and 3.0 ± 0.7 pg at day 28) and for healthy cartilage (1.6 ± 0.6 pg/mL at day 7 and 2.6 ± 1.4 pg at day 28). We did not detect IL-6 at days 7 and 28 in cell-free OA cartilage explant supernatants and at day 28 in cell-free healthy cartilage explant supernatant, whereas at day 7 cell-free healthy cartilage supernatant contained some IL-6 (103 ± 292 pg). IL-8 in supernatant of healthy cell-free cartilage explants was 4,347 ± 1,604 pg/mL at day 7 and 527 ± 667 pg at day 28. Cell-free OA cartilage explant supernatants contained IL-8 (2,905 ± 1,953 pg/mL) at day 7 and no IL-8 at day 28 (Figure 7). We also quantified IL-10 and tumor necrosis factor-alpha (TNF-α) at days 7 and 28 in supernatants of mono- and cocultures, but their concentration was close to the ELISA detection limit (data not shown). Soluble collagen concentration in the supernatants of the different culture regimens was measured with a hydroxyproline assay, but no differences were detectable (Additional file 2: Figure S2). GAG concentration in cell culture supernatants was significantly upregulated in all coculture regimens compared with monocultures at days 7 and 28 (Figure 6D). We demonstrated that, in cartilage explant culture supernatants which were cultured without cells, GAG concentration (43 ± 23 μg/mL) was comparable to soluble GAG measured in coculture supernatants (data not shown). In contrast, the concentrations of total soluble collagens (hydroxyproline assay) (Additional file 2: Figure S2) and fibronectin fragments (ELISA) (data not shown) in culture supernatants were not altered in the presence of cartilage explants.

We detected high concentrations of pro-inflammatory cytokines IL-1β, IL-6, and IL-8 and soluble GAG in supernatants of co- and tri-cultures but not of monocultures. In contrast, concentrations of IL-10, TNF-α, fibronectin, and total collagen were similar in co-, tri-, and monocultures.

### Stimulation of fibrin gel-embedded monocultured cells with interleukin (IL)-1β, IL-6, or IL-8

**Figure 8:**
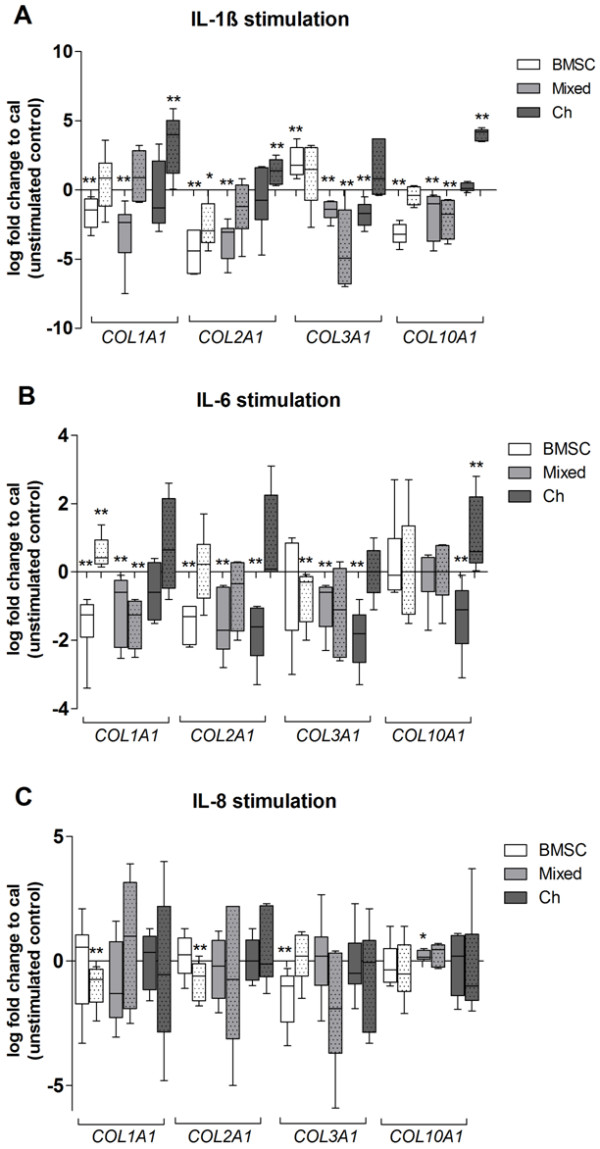
**Stimulation of fibrin gel monocultures with interleukin-1 beta (IL-1β), IL-6, or IL-8.** Collagen gene expression of **(A)** IL-1β, **(B)** IL-6, and **(C)** IL-8 stimulated monocultured cells. Bone marrow-derived stem cells (BMSCs) (white bars), mixed cultures (BMSCs plus chondrocytes 1:1, light grey bars), or chondrocytes (dark grey bars) were embedded in fibrin gel and were stimulated with 5 ng/mL IL-1β, 5 ng/mL IL-6, or 10 ng/mL IL-8 daily for 7 days in chondrogenic medium containing dexamethasone and transforming growth factor-beta 3 (TGF-β3). Cultures were analyzed for gene expression of *COL1A1*, *COL2A1*, *COL3A1*, and *COL10A1* at day 7 (blank bars) and day 28 (bars with dots). Results are presented as mean with standard deviation. *P <0.05; **P <0.01; ***P <0.001. N = 7. Cal, calibrator which represent the unstimulated monocultures; Ch, chondrocytes.

To determine whether stimulation of monocultures with IL-1β, IL-6, or IL-8 affects gene expression in a way similar to the stimulation observed in cocultures, mRNA expression of *COL1A1*, *COL2A1*, *COL3A1*, and *COL10A1* was analyzed at days 7 and 28. We observed a significant inhibition of *COL1A1* gene expression in IL-1β-stimulated BMSCs and in mixed cultures (day 7) and additionally a significant upregulation of *COL1A1* in chondrocytes (day 28) in comparison with unstimulated controls. A decrease of *COL2A1* gene expression was detected in IL-1β-stimulated BMSCs (days 7 and 28) and mixed cultures (day 7). In chondrocytes (day 28), a significant upregulation of *COL2A1* gene expression compared with unstimulated controls was observed. In BMSC cultures, we detected a significant upregulation of *COL3A1* gene expression at day 7. In contrast, in mixed cultures (days 7 and 28) and in chondrocytes (day 7), gene expression of *COL3A1* was significantly reduced. A decrease of *COL0A1* gene expression was detected in IL-1β-stimulated BMSC (day 7) and mixed (days 7 and 28) cultures. In chondrocytes (day 28), a significant upregulation of *COL10A1* gene expression compared with unstimulated controls was observed (Figure 8A).

IL-6 stimulation of BMSC (day 7) and mixed (days 7 and 28) cultures led to downregulation of *COL1A1* gene expression compared with controls. In contrast, BMSCs on day 28 revealed a significant upregulation of *COL1A1* mRNA level, whereas chondrocytes were not affected. *COL2A1* gene expression in all three culture conditions at day 7 was significantly downregulated by stimulation with IL-6. *COL3A1* expression was downregulated in BMSCs (day 28), mixed cultures (day 7), and chondrocytes (day 7) in comparison with respective unstimulated controls. Gene expression of *COL10A*1 was affected only in chondrocytes, where it was significantly lower at day 7 and significantly higher at day 28 in comparison with the unstimulated control (Figure 8B).

Stimulation with IL-8 induced a decrease in *COL1A1* and *COL2A1* gene expression of BMSCs on day 28, whereas *COL3A1* gene expression was significantly reduced at day 7. IL-8-stimulated mixed cultures and chondrocytes remained unchanged. An upregulation of *COL10A1* gene expression in comparison with controls was observed at day 7 in mixed cultures (Figure 8C).

Overall, we observed inhibitory effects of IL-1β and IL-6 on collagen gene expression of BMSC and mixed cultures, whereas collagen gene expression in chondrocytes was partly upregulated at the end of culture. IL-8 effects were negligible.

## Discussion

For long-term repair and regeneration of traumatic focal cartilage defects, chondrocytes and sometimes BMSCs are implanted at the site of injury; however, not much attention has been paid to microenvironmental effects of neighboring cartilage/subchondral bone. Induction of mRNA and protein expression of several chondrogenic markers was reported by treatment of BMSCs with growth factors like TGF-β [26] and also by paracrine factors released from cocultured articular cartilage or chondrocytes [12,14,27].

To identify conditions which favor proper ECM formation and chondrogenic differentiation and stability, we established a novel co- and tri-culture system where we used cartilage tissue explants for coculturing with cells. Our study demonstrated that coculture with these OA cartilage explants influences gene expression and biosynthesis of collagens I, II, III, and X in our two coculture regimens, including BMSCs and chondrocytes, and mixed tri-cultures of BMSCs together with chondrocytes. Overall, we observed an inhibition of gene expression of all collagens investigated in all culture regimens in the presence of cartilage explants, and this indicates that no collagen type-specific factor (that is, transcription factor) is responsible for inhibition of collagen expression on a gene level. Notably, inhibition of collagen protein biosynthesis was cell type-dependent; that is, collagen I, II, and III production was not affected in chondrocytes, whereas collagen I and II protein was reduced in BMSCs and collagen I and III in mixed culture setups. However, the effect must have some specificity to collagens as we did not observe alterations in proteoglycan (GAG) synthesis and deposition into the ECM in cocultures compared with monocultures.

Tissue matrix homeostasis underlies a delicate balance of matrix turnover and in parallel formation of new matrix. Therefore, we addressed the question of whether degradation processes contribute to a reduction in collagen content in cells and ECM in the presence of cartilage explants. We were unable to detect changes in soluble collagen concentration in the supernatants of the different culture regimens. We assume therefore that reduced collagen content is not due to increased degradation but presumably *a priori* to decreased biosynthesis.

With respect to GAG production and content, we did not find a difference between mono- and co-/tri-cultures. However, soluble GAG fragments were increased in the supernatant of cartilage cocultures, indicating increased degradation of aggrecan and other proteoglycans [28,29] which would support the increase in hydraulic matrix permeability. We assume that degraded GAG fragments in the supernatant were released by co- and tri-cultured cells and by OA cartilage explants in an additive way. Therefore, we assume no induction of GAG release in cocultured chondrocytes and BMSCs. However, these soluble GAG fragments might induce degradation of other ECM components as it is known that GAG fragments released from articular cartilage precede catabolism of collagen II [30,31]. It might be beneficial with respect to chondrogenic differentiation of BMSCs that cartilage-derived factors suppress collagen I and III gene expression and biosynthesis. Collagen I, a marker for dedifferentiation [32,33], and collagen III, a mesenchymal collagen, highly expressed in undifferentiated BMSCs [34,35], were repressed in BMSC cocultures and mixed tri-cultures. In all co- and tri-culture regimens of our study, gene expression of the hypertrophic marker *COL10A1*[36] was profoundly downregulated in the presence of articular cartilage. This phenomenon appears not to be restricted to OA cartilage but was described also for healthy cartilage cocultured with BMSCs [12], indicating that cartilage coculture can have a protective effect and can promote stability of the chondrogenic versus hypertrophic phenotype. However, we did not find corresponding collagen X immunostaining in cell fibrin gels—except some reactivity in BMSC monocultures—indicating no increased hypertrophic activity in monocultures. Collagen II, a positive chondrogenic differentiation marker [37,38], was also inhibited in cocultured BMSCs but remains more or less unchanged in mixed tri-cultures and chondrocyte cocultures. Our data hint to soluble OA cartilage released factor(s) which contribute to reduced chondrogenic differentiation capacity of BMSCs, whereas chondrocytes as fully differentiated cells are less responsive to these instructions. Although many studies have examined effects of culture medium supplemented with well-defined chondrogenic factors on differentiation capacity of BMSCs, only few have addressed the influence of OA-affected cartilage ECM and OA chondrocyte-conditioned culture medium on matrix formation of cocultured cells. In the present study, we thus analyzed pro-inflammatory factors known to be produced by OA cartilage in culture medium supernatants. Cytokines like IL-1β, IL-6, and IL-8 have been shown to be secreted from OA chondrocytes and are considered to contribute to OA pathogenesis [39]. In our experimental setup, we observed that coculture with OA cartilage clearly increased the level of pro-inflammatory cytokines IL-1β, IL-6, and IL-8 in supernatants of all culture regimens. IL-1β is a known suppressor of *COL2A1* gene expression in OA cartilage and thus might be responsible for reduced *COL2A1* gene expression in cocultures compared with monocultures [40]. IL-6 and IL-8 were significantly increased at day 7 and decreased on day 28 in cartilage cocultures. It is reported that traumatic injury of joints causes immediate release of pro-inflammatory cytokines like IL-1β, IL-6, and IL-8 in the synovial fluid and increases the risk of developing OA [41,42]. OA joints contain more IL-6 than unaffected joints in synovial fluid, and OA chondrocytes produce more IL-6 during cartilage regeneration than healthy chondrocytes. This high local concentration could affect regeneration processes and support a modest anabolic role for IL-6 in cartilage matrix production [43].

Stimulation of monocultures with IL-1β and IL-6 resulted in an overall reduced gene expression response in BMSC and mixed cultures; however, IL-1β increases *COL1A1*, *COL2A1*, and *COL10A1* gene expression in chondrocytes at the end of the culture period. This could be interpreted as an anabolic effect of IL-1β on OA chondrocytes, which are known to exhibit a phenotype switch toward repair mode in order to replace destroyed cartilage accompanied by increased collagen synthesis. We observed a profound increase in supernatants of cocultures for IL-8; however, stimulation of monocultures with IL-8 resulted in only modest changes in collagen gene expression. Possibly, IL-8 induces other metabolic activities in cells (that is, cell migration) as IL-8 is known as a chemotactic chemokine which initiates migration of chondrocytes and MSCs [44].

Most important for successful cartilage defect repair are biomechanical properties of the newly formed tissue because aberrant mechanical loading is a major OA-promoting factor leading to alteration in chondrocyte metabolism [45]. It is suggested that OA cartilage has a different sensing of the mechanical environment compared with normal cartilage [46]. Additionally, resident cell populations show an inappropriate response to mechanical stress which might be important in disease progression [47]. The very specific mixture of fluid (water) and ECM (collagens and proteoglycans) provides its visco-elastic properties for efficient and equal distribution of load implemented on hyaline cartilage. Once under stress, the load is initially carried by interstitial fluid, which then is displaced from the tissue and leaves a less hydrated ECM, which carries the load after water is exuded from the tissue. Both fluid and ECM determine biomechanical properties of articular cartilage; therefore, cartilage is best seen as a biphasic structure [48]. In this line, we revealed that coculture with OA cartilage influences biomechanical properties of newly formed matrix of cocultured cells. Young’s modulus and aggregate modulus at equilibrium were reduced particularly in cocultures of BMSCs and tri-cultures of mixed cells, whereas hydraulic permeability was increased. Our results suggest that coculture of BMSC and mixed cultures with articular cartilage leads to a reduced capacity of matrix to withstand mechanical stress (that is, load and increased porosity and fluid exchange). In contrast, cocultured chondrocytes showed a trend to increased Young’s modulus (at 40% to 50% strain), which indicates a superior matrix during unconfined compression compared with BMSCs and tri-cultures. Overall, cocultured cells seem to produce a highly porous matrix impairing the ability to carry loads. As cartilage-derived factors inhibited *de novo* collagen protein expression in cocultures, GAG synthesis remained unchanged. Even though soluble GAG was increased in coculture supernatants indicating proteoglycan degradation, this increase is presumably due to an additive effect of both cocultured cells and cartilage explants. Therefore, we assume that alterations in biomechanical properties are due mostly to changes in the fibrillar collagen network; however, loss of proteoglycans from the extrafibrillar matrix is likely to contribute. Reduced biosynthesis of collagens impairs the formation of stable proper interconnected collagen networks which crucially affects the structural integrity of the ECM, leading to inferior mechanical and biochemical properties.

We have included a mixed coculture regimen (tri-culture) into our experimental setup to determine whether OA chondrocytes isolated from their pathological tissue environment and cultured together in close contact with BMSCs have different effects than chondrocytes which reside inside their native environment [14]. In addition, we wanted to determine whether differentiated chondrocytes which are in direct cell-to-cell contact with undifferentiated BMSCs ameliorate or augment inhibitory effects of cartilage explants on BMSCs. Overall, we observed that collagen gene and protein expression and biomechanical properties of tri-cultured mixed populations resemble more closely cocultured BMSCs. Also, stimulation of mixed monocultures with IL-1β and IL-6 resulted in changes in gene expression of collagens which were similar to the changes for BMSC monocultures. This observation is in contrast to other studies which report anabolic effects of cocultured chondrocytes on BMSCs with respect to biomechanical properties, collagen, and GAG production [15,16]. On the other hand, Giovanni and colleagues [17] report that influence of cocultured chondrocytes was restricted to early signs of neochondrogenesis and did not prevent hypertrophy of BMSCs. We thus suggest that chondrocytes in the mixed cultures do not seriously alter BMSC metabolism in tri-cultures with cartilage explants and that response of mixed cultures to cartilage explants resembles that of cocultured BMSCs rather than that of cocultured chondrocytes.

## Conclusions

We established a novel coculture system where chondrocytes and BMSCs are cocultured with cartilage explants (including cells and matrix). With respect to our data, we suggest that these OA cartilage explants have an inhibitory effect on the production of all collagens analyzed but that proteoglycan production is not affected in cocultured BMSCs and chondrocytes. Reduction of collagen synthesis impairs biochemical and biomechanical properties of the newly formed ECM derived from cocultured cells. This prevents presumably mainly the formation of a stable and well interconnected fibrillar collagen network which eventually facilitates degradation of proteoglycans. We suggest that soluble factors, including IL-1β, IL-6, IL-8, and GAGs, released from OA cartilage, mediate these effects partially. OA cartilage seems to provide inhibiting signals for chondrogenic differentiation of undifferentiated BMSCs and formation of a stable and mechanical stress-resistant ECM. This observation will have an impact on future therapeutic strategies to halt or even reverse OA progression. In consequence, microenvironmental factors from surrounding cartilage tissue need to be taken into serious consideration when trying to implement novel OA treatment strategies (that is, implanting cells into lesions and fissures prevalent in late stages of OA cartilage).

## Abbreviations

3D: three-dimensional; BMSC: bone marrow-derived stem cell; COL10A1: collagen X A1 (gene); COL1A1: collagen I A1 (gene); COL2A1: collagen II A1 (gene); COL3A1: collagen III A1 (gene); DMMB: dimethylmethylene blue; ECM: extracellular matrix; ELISA: enzyme-linked immunosorbent assay; GAG: glycosaminoglycan; h_0_: initial height; IL: interleukin; LDH: lactate dehydrogenase; MSC: mesenchymal stem cell; OA: osteoarthritis; PBS: phosphate-buffered saline; PCNA: proliferating cell nuclear antigen; PCR: polymerase chain reaction; PFA: paraformaldehyde; qPCR: quantitative polymerase chain reaction; RT: room temperature; TGF-β: transforming growth factor-beta; TNF-α: tumor necrosis factor-alpha.

## Competing interests

The authors declare that they have no competing interests.

## Authors’ contributions

ML had carried out acquisition of all data and contributed substantially to analysis and interpretation of the data. AS and LD contributed substantially to the biomechanical testing, analysis, and data interpretation of such. H-RS and PA contributed substantially to data acquisition. AI and JG were involved in revising the manuscript critically for important intellectual content. SG carried out the design and coordination of the study and contributed substantially to drafting and revision of the manuscript critically for important intellectual content. All authors read and approved the final manuscript.

## Supplementary Material

Additional file 1: Figure S1 Vitality and percentage of proliferating cell nuclear antigen (PCNA)-positive cells of fibrin gel-embedded monocultures and coculture setups. Vitality of **(A)** bone marrow-derived stem cell (BMSCs), **(B)** mixed cultures (BMSCs and chondrocytes in a ratio of 1:1), and **(C)** chondrocytes was determined in monocultures (F) and cocultures with articular osteoarthritis (OA) cartilage (FC) kept in chondrogenic medium. Content of lactate dehydrogenase (LDH) was quantified in the supernatant of days 7, 14, 21, and 28 and compared with controls (high control = all cells in a fibrin gel were lysed; low control = spontaneous cell death of an equivalent cell amount in monolayer). Owing to high inter-experimental variability, we have calculated the raw data as percentage of control per individual experiment. **(D)** Mitotic activity of BMSCs (white bars), mixed cultures (light grey bars), and chondrocytes (dark grey bars) was determined in monocultures (F) and cocultures with articular OA cartilage (FC) kept in chondrogenic medium. PCNA-positive stained cell nuclei were counted at days 7 and 28, and percentage of positive cells to total cell number was calculated. Results are presented as mean with standard deviation. *P <0.05; **(A-C)**: n = 4; **(D)**: n = 5. Ch, chondrocytes. Click here for file

Additional file 2: Figure S2 Hydroxyproline concentration in supernatants. Culture supernatants of bone marrow-derived stem cells (BMSCs) (white bars), mixed cultures (BMSCs and chondrocytes in a ratio of 1:1, light grey bars), or chondrocytes (dark grey bars) monocultured (F, bars with pattern) or cocultured with osteoarthritis (OA) cartilage (FC, blank bars) were analyzed at days 7 and 28. Total soluble collagen in the supernatant was determined by using a hydroxyproline assay. Results are presented as mean with standard deviation. *P <0.05; n = 6. Ch, chondrocytes. Click here for file
