# Supplementary figures and images for: Osteoarthritic cartilage explants affect extracellular matrix production and composition in cocultured bone marrow-derived mesenchymal stem cells and articular chondrocytes
Source: Stem Cell Res Ther. 2014 Jun 10;5(3):77. doi: 10.1186/scrt466 (PMC4097830; doi:10.1186/scrt466)

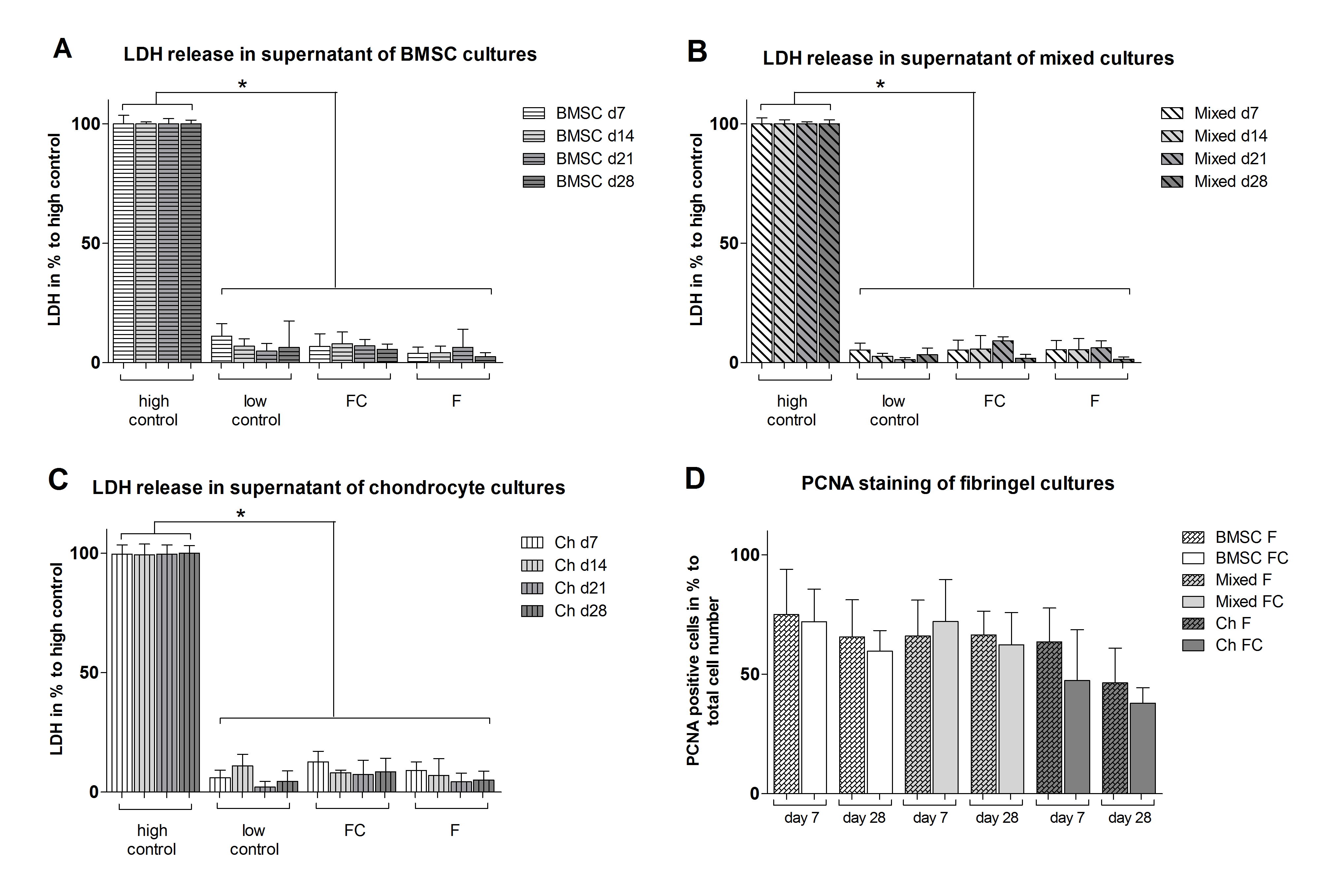

Supplement: Additional file 1: Figure S1 — Vitality and percentage of proliferating cell nuclear antigen (PCNA)-positive cells of fibrin gel-embedded monocultures and coculture setups. Vitality of (A) bone marrow-derived stem cell (BMSCs), (B) mixed cultures (BMSCs and chondrocytes in a ratio of 1:1), and (C) chondrocytes was determined in monocultures (F) and cocultures with articular osteoarthritis (OA) cartilage (FC) kept in chondrogenic medium. Content of lactate dehydrogenase (LDH) was quantified in the supernatant of days 7, 14, 21, and 28 and compared with controls (high control = all cells in a fibrin gel were lysed; low control = spontaneous cell death of an equivalent cell amount in monolayer). Owing to high inter-experimental variability, we have calculated the raw data as percentage of control per individual experiment. (D) Mitotic activity of BMSCs (white bars), mixed cultures (light grey bars), and chondrocytes (dark grey bars) was determined in monocultures (F) and cocultures with articular OA cartilage (FC) kept in chondrogenic medium. PCNA-positive stained cell nuclei were counted at days 7 and 28, and percentage of positive cells to total cell number was calculated. Results are presented as mean with standard deviation. *P <0.05; (A-C): n = 4; (D): n = 5. Ch, chondrocytes. [file scrt466-S1.tiff]

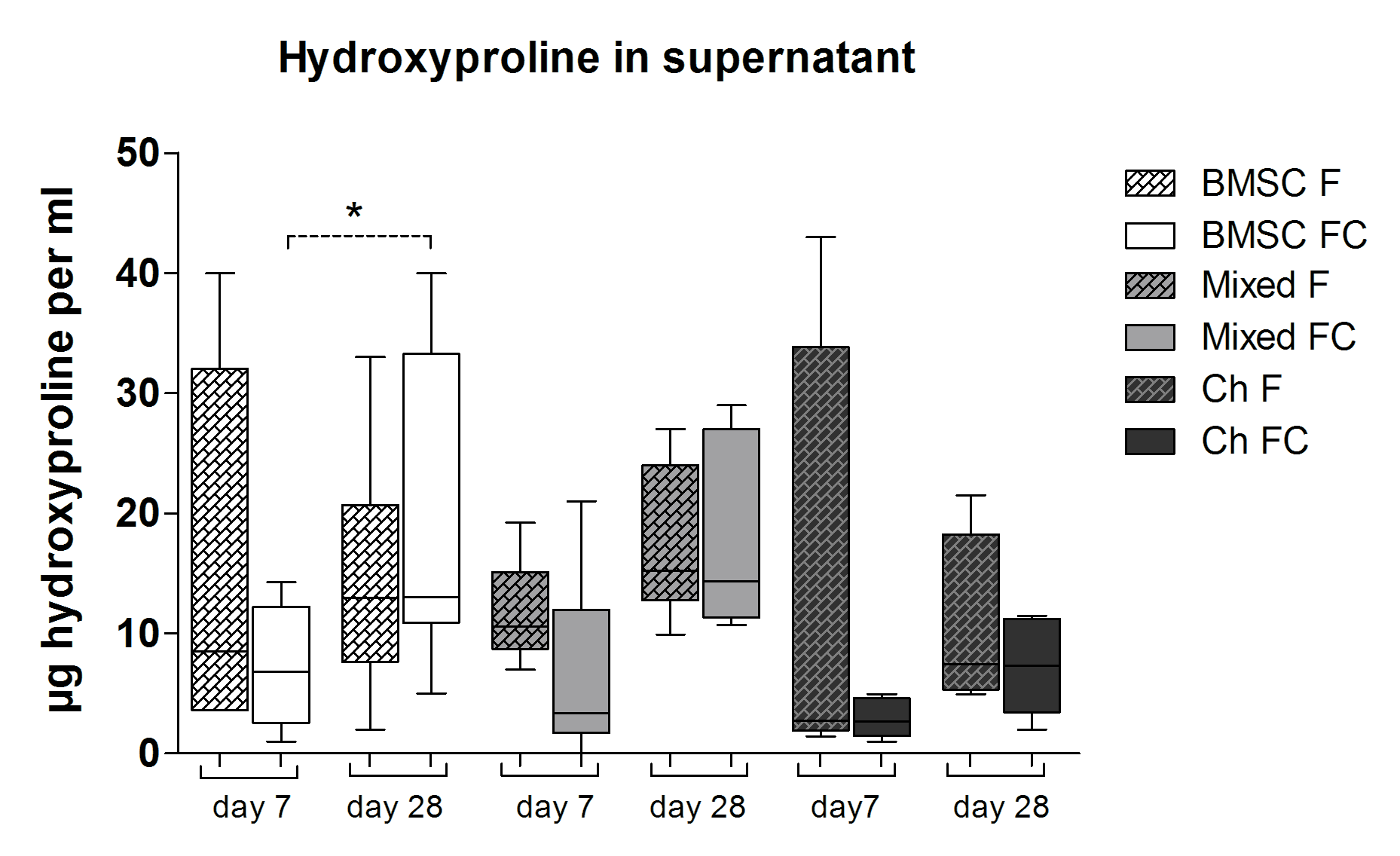

Supplement: Additional file 2: Figure S2 — Hydroxyproline concentration in supernatants. Culture supernatants of bone marrow-derived stem cells (BMSCs) (white bars), mixed cultures (BMSCs and chondrocytes in a ratio of 1:1, light grey bars), or chondrocytes (dark grey bars) monocultured (F, bars with pattern) or cocultured with osteoarthritis (OA) cartilage (FC, blank bars) were analyzed at days 7 and 28. Total soluble collagen in the supernatant was determined by using a hydroxyproline assay. Results are presented as mean with standard deviation. *P <0.05; n = 6. Ch, chondrocytes. [file scrt466-S2.tiff]
